# Modelling the future climate impacts on hydraulic infrastructure development in tropical (peri-)urban region: Case of Kigali, Rwanda

**DOI:** 10.1016/j.heliyon.2024.e27126

**Published:** 2024-02-24

**Authors:** Parfait Iradukunda, Erastus M. Mwanaumo, Joel Kabika

**Affiliations:** a Department of Civil and Environmental Engineering, School of Engineering, University of Zambia, PO. Box 32379 Lusaka, Zambia; b Department of Civil Engineering, College of Science, Engineering, and Technology, University of South Africa, PO. Box 392, Pretoria, South Africa

**Keywords:** Nyabugogo river catchment, Climate projection, Hydraulic structures, Hydrologic model, Bridge hydrodynamic, Hydrodynamic model, Climate impacts and adaptation

## Abstract

The current global climate has shown a significant change, mostly resulting from human-induced activities. Frequent experiences of extreme rainstorms, deadly landslides, and floods followed by the destruction of roads, bridges, drainage, buildings, agriculture, and other infrastructures have been appearing across the globe along with extensive socio-economic effects including human lives losses whereby tropical Africa is among the greatly affected regions. Several studies in the region acclaim the increase of climate-related extremes due to a gradual climate variation. Hence, this study aimed to evaluate how existing water structures might respond to the future climate, which is getting more severe and frequent in the region. The study was conducted on the Nyabugogo River catchment (NRC), covering a huge part of the Kigali metropolitan area. It was carried out through a downscaled global climate model (CMIP6 GCM) projection coupled with a joint SWAT + hydrological model and HEC-RAS hydrodynamic simulation. The study showed that the annual precipitation in Kigali might keep increasing, resulting in increased risks of extreme weather events. The study identified up to 38% (+514.9 mm) annual precipitation increment, which resulted in more than a doubled flow rate (+28.0 $m^{3}/s$) increment by the end of the century under a high greenhouse gas emission scenario (ssp585). As a result, hydrodynamic simulations revealed that the Bridge-1 in NRC might fail to accommodate the 50-year return peak storm under ssp585. Henceforth, there is a need to adopt high GHG emission scenarios in critical infrastructure development. Further, enforcing green-grey infrastructures in flood risk-low resilient areas is recommended to improve climate resilience. Thus, the results of this study might prove useful in climate-resilient infrastructure development and other pre-emptive adaptation practices, most importantly building anticipated resilience against climate-related hazards.

## Highlights

• Impact of future climate on water infrastructure in the tropical urban region.
• Stage-discharge Rating Curve (SRC) is ideal for poor or less gouged catchments.
• Precipitation magnitude in a cool-dry season and long rainfall season from NRC might become quite close by the mid-century.
• Peak storms in NRC might double by the end of the century under ssp585.
• The Bridge-1 and 4 might fail to accommodate the 50-year return peak flow under ssp585.

## 1 Introduction and background

The uncertainty of climate change keeps raising challenges in water-related infrastructure development [1]. As extreme climatic events become more severe and frequent, it raises concern about which level of our infrastructure is prepared to deal with these hydroclimatic changes [2]. From the so-called “stationary climate assumption” [2], the bridges and culverts' hydrologic designs are often based on historical climate records for the essence of resisting the regular n-year flood events assuming the rainfall is roughly constant with insignificant long-term climate impacts on rainfall intensities variation over the time. For example, a river crossing highway bridge might be stationarily designed to pass the 100-year flood [3]. However, these climate patterns are dramatically changing, and these changes result in regional rainfall intensity variations altering to the climatic extremes [4,5]; hence, that bridge might fail to withstand that 100-year flood, a concept termed “nonstationary climate assumption” [[1], [2], [3], [4],^6^]. Global warming increases atmospheric humidity and leads to increased precipitation and extreme flood risk [7,8]. As emphasised in much literature [6], the nature of the climate will keep dying due to the substantial anthropogenic impacts altering to extremes if there are no rigorous mitigation measures adopted. In case such mitigations are not achieved, the rates of impacts are expected to accelerate more, although the exact magnitude might remain uncertain [9]. Moreover, the manifestation and effects of climate change vary from one place to another [10]. Therefore, appropriate climate status and adaptations are studied according to the regions.

The current global infrastructures face multiple challenges, naturally and unnaturally related, but often climate change results [6]. Several works of literature averred that extreme climate change implications on infrastructure might rise more severely and frequently [2]. Leaving out these changes during the designs might lead to inadequacy, resulting in under-designed structures that fail too often or over-designed structures that are economically inefficient. Severe flood water application can lead to the bridge's total failure [3], plus direct health and socio-economic effects like traffic inaccessibility, damages, accidents, and human life losses. Further studies are recommended based on recorded and projected changes and suggested to be incorporated in future structure construction to minimise the risks during the structure lifespan [[11], [12], [13], [14], [15], [16], [17], [18], [19], [20]]. Among them, some emphasised the necessity of regularly updating the standard codes of practice to evaluate, attribute, and cope with the updates within the changing climate conditions, such as adjusting the flood design factors and rainfall estimations [12,17,20]. Meanwhile, multiple studies have tried to incorporate the changes through the IDF Curves [3,4,21,22].

Extreme rainstorms coupled with deadly landslides have become severe and frequent in Rwanda. Several extreme weather incidences followed by the destruction of buildings, roads, bridges, electric systems, agriculture, and other infrastructures have been reported, along with extensive effects on economic and public health sectors, including human life losses. According to the MIDIMAR report and the media, about 14, 10, 14, and 17 people lost lives in 2006, 2010, 2011, and 2012, respectively. More than 72, 11, and 131 people died over heavy rainfall coupled with massive landslides in 2020, 2022, and 2023, respectively [[23], [24]]. Hundreds of houses were destroyed, and thousands of people were displaced, forcing them to seek refuge. Storms coupled with landslides damaged roads and bridges and many more thousands of hectares of farmlands, especially in the North-western region of the country [25]. Referring to the studies conducted in the region [[24], [26], [27]], up to 29% of rainfall intensity is expected to increase with +60% frequency by the end-century. Such rainstorm increments might eventually have a significant impact on infrastructure and hydrologic regime. Besides, Nyabugogo and some other areas within the Kigali metropolis were reported to have been experiencing severe flooding incidents and drainage overflows, such as the bridges along the Mpazi drainage channel.

Throughout the review, a number of studies have evaluated the impacts of future climate on the hydrologic regime in the region and identified a significant variation [24,28,29]. However, no study was identified to have evaluated weather-related bridge and culvert hydrodynamics. Consequently, the impacts that might result remain uncertain. It drove our attention to assessing the future climate impacts on water infrastructure. Hence, this work aims to examine how the current hydraulic structures might respond to future climate variability, which is getting more severe and frequent in the region. Thus, the results of this study might prove helpful in climate-resilient infrastructure development and other pre-emptive adaptation practices, most notably building anticipated resilience against weather-related hazards.

## 2 Research methodology

### 2.1 Study area

**Fig. 1:**
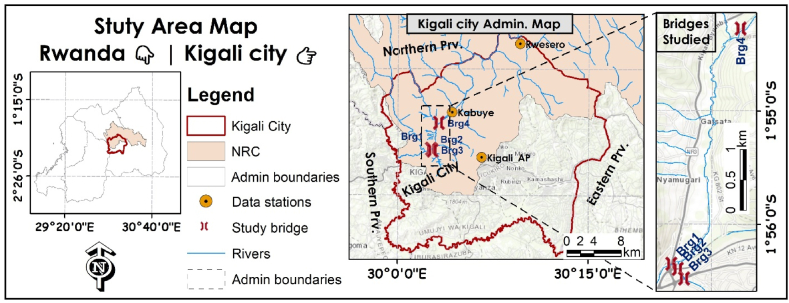
Map of Nyabugogo River Catchment in the Kigali metropolitan area.

**Fig. 2:**
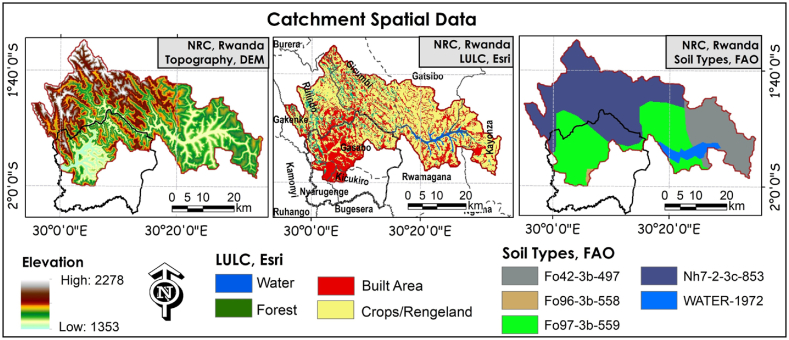
Spatial data identification (Topographic terrain, LULC, and Soil categories) in the catchment.

Rwanda is a landlocked country in the central African region with a temperate, tropical highland climate. Kigali is the capital city of Rwanda, lounges in a highly sloped region with numerous hills, ridges, and valleys. It is located approximately in the centre of the country (Fig. 1). Rwanda, as a densely populated area with growing urbanisation, might be highly vulnerable to changes in climate and land use/cover. Urbanisation and population density in Kigali city have been gradually increasing from 6000 in 1960 to over 200,000 in the early 1990s. The population of Kigali city was approximately 1.2 million by 2013 and is expected to increase from 4 to 5 million in the next 25–30 years [30]. The topmost average monthly rainfall varies over a range of 200–250 mm in April and November, which are the peaks of the two rainy seasons in the country. The rest of the regions fall into the range below 200 mm. June, July, and August are the warm-dry seasons marked with monthly precipitation lesser than 50 mm. The annual maximum and minimum temperatures were 27.5 °C and 16.5 °C by 2010, respectively, with an increase of 0.35 °C per decade [24,26,31]. This study was conducted specifically on the bridge structures under the Nyabugogo River Drainage Basin (NRDB), which occupy a big part of Kigali's urban settlement where the most reported flooding areas, such as Nyabugogo, and the drainage overflows such as the bridges along Mpazi drainage channel are found [28,29,32]. Meanwhile, the fact that a huge part of the Kigali metropolitan area is in a lower altitude area of NRDB (Fig. 2) makes it more subject to the floods induced by heavy rainfall experiences from this basin.

### 2.2 Types and sources of dataset

The temperature and precipitation data for the three main stations (Fig. 1) from the 1981 to 2021 period were obtained from the Rwanda Meteorology Agency and prepared and processed accordingly [24,33]. The famous Pettitt's test, Buishand's Range (BHR), and Standard Normal Homogeneity Test (SNHT) were used for weather data homogeneity and validity analysis [24,34]. The future climate analysis was conducted using NASA Earth Exchange Global Daily Downscaled Projections CMIP6 dataset ensembles (NEX-GDDP-CMIP6) [35,36]. The bias correction spatial disaggregation (BCSD) algorithm was used to produce the NEX-GDDP datasets [37]. The CMIP6 GCM was developed in support of the Intergovernmental Panel on Climate Change, Sixth Assessment Report (IPCC AR6) [38]. Observed streamflow data and stage levels were obtained from the Rwanda Water Board water portal. The study used the SWAT + model, which simultaneously uses solar radiation, wind speed, and relative humidity, which were simulated from the SWAT global weather database. The spatial data, i.e., Digital Elevation Model (DEM), Land Use/Cover (LULC), and soil types, were spatially collected from USGS Landsat imagery, Esri, and FAO/UNESCO, respectively. The study identified multiple bridges in the catchment, and the most vulnerable were screened based on (1) the areas frequently reported (in the literature) to have been experiencing flooding incidences, (2) the flow accumulation results, (3) the site visits took place in the rainy seasons, where the marks of previous or highest flood level are left to the bridge opening and river banks, and (4) information obtained from the frequent road users (local taximen). Thereafter, hydraulic measurements were physically measured during the site visits.

### 2.3 Climate projection and bias correction

The NEX-GDDP-CMIP6 dataset includes bias-corrected and downscaled projections for different climatic patterns over different Shared Socio-economic Pathways (SSP) scenarios assuming higher mitigation to a no-change scenario as SSP126, SSP245, SSP370, and SSP585, respectively, for low, intermediate, high, and the most extreme Greenhouse Gas (GHG) concentration scenarios for periods 1950 to 2100 over 0.25° (∼27 km × 27 km) spatial resolution [35,36,39]. Since a multi-model ensemble (MME) is acclaimed to be more reliable than the best single model [40,41], the study was conducted from the projected climate for the mid-term and long-term, i.e., the 2050s (2051–2060) and 2090s (2091–2100) relative to the current period as reference, the 2010s (2011–2020) for scenarios imposed by medium and high GHG emissions scenarios (SSP245 and SSP585) derived from five model ensemble (Table 1) which were selected based on all SSPs availability and the performance in various works of literature [39,41,42]. This study used the statistical bias correction and downscaling approach with 41 years of overlapping observations. The statistical bias correction reanalysis was conducted using linear scaling, distribution mapping, and local intensity scaling methods for precipitation plus variance scaling for temperature [43,44].

**Table 1:** Detailed information on selected CMIP6 GCMs.

Model Name	Institute & Origin	Resolution	Reference
ACCESS-CM2	Commonwealth Scientific and Industrial Research Organization (CSIRO), Australia	1.25° × 1.87°	[45]
CanESM5.0.3	Canadian Centre for Climate Modelling and Analysis, Environment and Climate Change Canada, Canada	2.8° × 2.8°	[46]
CNRM-CM6-1	Centre National de Recherches Meteorologiques and Centre France Europeen de Recherches et de Formation Avancee en Calcul Scientifique, France	1.4° × 1.4°	[47]
MPI-ESM1-2-HR	Max Planck Institute for Meteorology, Germany	0.93° × 0.93°	[48]
NorESM2-MM	Norwegian Climate Centre, Norway	0.93° × 1.25°	[49]

### 2.4 Hydrologic model development

#### 2.4.1 Model setup

The DEM, LULC, and soil characteristics are the primary spatial data inputs in hydrologic model processing. The terrain topography was identified using 30-m resolution DEM data obtained from the USGS portal. The LULC was processed based on Esri 10-m resolution LULC classes, while the catchment soil characteristics were processed based on FAO/UNESCO soil groups. Five soil groups were identified in the basin (Fig. 2). The study used a new generation of the SWAT model named the SWAT + model (version 60.5). Hydrologic analysis was conducted using the QSWAT + interface. It is an integrated tool with a QGIS interface for generating the catchment flow direction, flow accumulation, and HRUs characteristics based on DEM, LULC, and soil characteristics. A prepared slope map classified into different slope classes-based DEM, the land use/cover containing four classes, the soil map with five soil groups, and their respective lookup tables were generated. About 235 channels with more than 5000 HRUs were generated in the catchment. The SWAT + Editor was hence used to run the model based on more than 100 years of climatic factors (temperature, precipitation, solar radiation, humidity, and wind direction). After running the model, the SWAT + Toolbox was used for sensitivity analysis and running automatic calibration and model validation.

#### 2.4.2 Integrated empirical methods in the models

Integrated Curve Number determination is essential for model routing and runoff formation. The models use integrated Natural Resources Conservation Service (NRCS), formerly termed the Soil Conservation Service-Curve number (SCS–CN), and Muskingum routing methods for runoff estimation. The SCS-CN method is used to model and analyse precipitation and infiltration loss directly to the surface runoff in the subbasins. It has three main parameters, which are Curve Number (CN), Initial Abstraction $\left(I_{a}\right)$, and the Percent Impervious Area in the basin. The CN is used in determining the soil potential maximum retention $\left(\prime \prime S^{\prime \prime }\right)$ as expressed within Eq. (2). The $\prime \prime S^{\prime \prime }$ value depends on the soil types, cover, and antecedent soil moisture condition. It is defined as a measure of the watershed ability to abstract and retain precipitation water [50]. The CN values range from zero (0%) for an infinitely abstracting with S=∞ to a hundred (100%) for zero potential retention (for water bodies or a case of perfect impervious surfaces and soils covered with near-zero infiltration rates). The higher the cover imperviousness or slower infiltration rates, the higher the CN [51]. To identify the wetness of the soil in the catchment, the SCS method subdivides the soil Antecedent Moisture Condition (AMC), currently termed Antecedent Runoff Condition (ARC), into three classes, which are ARC-I for dry soils, ARC-II for soil with average conditions, ARC-III for soil with sufficient rainfall occurred at least within the past 5–10 days maximum. The method also subdivides the soils into four groups (A, B, C, & D) based on infiltration capacity and some other characteristics [52]. Hence, the CN is identified based on the LULC respective to the ARC classes [[52], [53], [54]]. The criteria for each soil group and CN determination were plentifully addressed in Burke and Burke, [52], and Chow et al. [53].

The SCS-CN model assumes that accumulated Precipitation-Excess $\left(P_{e}\;\text{in}\;\text{mm}\right)\text{,}$ namely runoff, similarly depends on the cumulative rainfall, soil type, LULC, and the antecedent moisture conditions. However, it is directly proportional to the rainfall, (P). The initial abstraction amount is assumed to be proportional to the potential maximum retention $\left(I_{a}=\lambda S\right)$ [[52], [53], [54], [55]]. Therefore, the total accumulated precipitation runoff, $P_{e}$ is calculated following Eq. (1), where the SCS-CN method adopted empirical relation λ=0.2 as a standard value obtained throughout the results from many small-sized watershed experimental studies [53,56]. It was assumed that the runoff was produced after the initial abstraction of 20% of the potential maximum storage [50].

(1) $$P_{e}=\frac{{\left(P-0.2S\right)}^{2}}{P+0.8S},\text{for}\;P>\lambda S$$

(2) $$S=100\left(\frac{10}{\text{CN}}-1\right),\left(\text{in}\;\text{Inchs}\right)\;\text{or}\;S=254\left(\frac{100}{\text{CN}}-1\right),\left(\text{in}\;\text{mm}\right)$$

#### 2.4.3 Model calibration and validation

##### 2.4.3.1 developing the stage-discharge rating curve

In many African catchments with limited or incomplete current and historical gauged river flow data, a precise runoff simulation is intricate. However, a growing concern of model applications plus existing statistical techniques ascertain the possibility of feasible simulations. Due to the lack of observed time series flow data in the Nyabugogo catchment, a simple rating curve fitting method expressed in power form (Eq. (3)) and the datum correction determined using calibration coefficients (Eq. (4)) were applied to available water stage records at Nemba station (close to the catchment outlet) obtained from the Rwanda Water Board, water portal. The Stage-discharge Rating Curve (SRC) method is a statistical analysis technique to generate the discharge based on the stage levels and the field measurements [57,58]. A similar methodology was applied by Manyifika, [60], and Umugwaneza et al. [59]. With regards to Birbal et al. [61] and Kumlachew et al. [62], the studies vow an excellent statistical performance of SRC with more than 0.95 correlation in actual versus calculated discharge values. The SRC similarly presents a great performance on the precipitations versus the flow rate ratios in Manyifika, [60]. Since the model was aimed at bridge storm designs, the model calibration considered the daily peak stage level. The most critical bridges were selected based on the site visits that took place mostly in the rainy seasons.

(3) $$Q={C\left(h\pm a\right)}^{n}$$

(4) LogQ=LogC+n*log(h±a)

**Where:** where Q is the discharge; h is the stage level; C,a,andn are the calibration coefficients. C is the discharge when the effective depth of flow (h−a) is equal to 1; a is the gauge height of zero flow known as datum correction and determined either through trial and error, arithmetic procedure, or computer-based optimisation; n is the slope of the rating curve on logarithmic paper; (h−a) is the effective water depth on the control. When the exponent n approaches a 3/2 rating, it is also known as a Guglielmini rating curve [57,58].

##### 2.4.3.2 sensitivity analysis and accuracy evaluation

The model sensitivity was analysed on several hydraulic parameters suspected to be having a substantial impact on runoff quantity formation. Observation data were used to trace the model accuracy and calibration based on different standard statistical methods. Nash-Sutcliffe efficiency (NSE), Percent bias (PBias), and Root Mean Square Error (RMSE) were used for model quality evaluation (Table 2). The NSE is ideal in determining the relative variance magnitude compared to the observation data variance; PBIAS measured the model's average tendency to be larger or smaller over our observation discharge, while the RMSE measured the average error between predictions and observed series. The smaller the RMSE, the better the simulation. The above statistical goodness-of-fit indices are all most used and much-admired in various hydrological literatures [[63], [64], [65]]. The Swat + Toolbox used in this study performs calibration in two main stages: sensitivity analysis and automatic calibration. After sensitivity analysis, a rerun was assigned for automatic calibration with the newly sensitive parameters (Table 3). Auto-calibration runs multiple iterations through which the model is calibrated and validated till the model presents ideal results based on the performance indices or till the calibration shows no further change.

**Table 2:** Model performance validity and goodness-of-fit indices.

Coefficient	Formula	Optimal values
**NSE**	$\left[1-\frac{\sum_{t=1}^{T}{\left(Q_{m}^{t}-Q_{o}^{t}\right)}^{2}}{\sum_{t=1}^{T}{\left(Q_{o}^{t}-\bar{Q_{o}}\right)}^{2}}\right]$	NSE = 1, perfect match; ≈1, Optimal; 0.75 < NSE ≤1 Very Good; 0.6 < NSE ≤0.75 Good; 0.2 < NSE ≤0.6 Satisfactory; 0 ≤ NSE ≤0.2, Unsatisfactory
**PBIAS**	$\frac{\sum_{t=1}^{T}\left(Q_{o}^{t}-Q_{m}^{t}\right)*100}{\sum_{t=1}^{T}\left(Q_{o}^{t}\right)}$	PBias = 0–10%, Optimal, Negative, underestimation Positive, overestimation
**RMSE**	$\sqrt[{2}]{\left(\frac{\sum_{i=1}^{N}{\left({{\text{Actual}}_{i}-\text{Modelled}}_{i}\right)}^{2}}{N}\right)}$	RMSE = 0, perfect match, 0≤RMSE ≤1, Optimal

**Where:** N is the number of non-missing data, $Q_{o}^{t}$ is observed flow at a given timestep, $Q_{m}^{t}$, is a modelled flow at a given time step, while $\bar{Q_{o}}$ is the mean observed flow.

**Table 3:** Sensitivity analysis parameters and final calibration values.

S#	Calibration parameters	Sensitivity ratio		Changes (%)	
		ssp245	ssp585	ssp245	ssp585
**1**	cn2	0.1161	0.3669	20.0	17.4
**2**	cn3_swf	0.0309	0.8235	−20.6	−27.0
**3**	clay	0.0527	0.0183	21.3	8.5
**4**	chk	0.0199	−0.0313	−29.9	7.8
**5**	chw	0.0061	−0.0484	27.8	6.4
**6**	flo_min	0.2081	−0.0001	18.0	24.6
**7**	revap_min	0.2242	−1.3E-06	24.3	−29.7
**8**	canmx	0.0024	0.0861	−37.4	−39.2
**9**	alpha	0.0000	0.0052	0.0	0.5

### 2.5 Hydrodynamic model development

#### 2.5.1 Model setup and scenario design

The flood dynamics and hydraulic structure response to the climatic variation were analysed through the HEC-RAS model, whereby the digital terrain model (DTM) and peak flow data were the base data inputs to the model processing. The DTM is also an essential data input in all other flood inundation modelling. The DTM resolution has a significant effect on the model structure and performance, especially for the small-sized channels [[66], [67], [68]]. The study was initially conducted using a 10-m DTM resolution generated from ESA's Sentinel-1 radar imageries. However, since hydraulic modelling is sensitive to the DTM resolution, plus the fact the river width wasn't wide enough, this resolution was not as good as LiDAR or physical surveying (UAVs or GNSS). Therefore, the study had to exercise the GNSS survey (global navigation satellite system) to correct some errors resulting from poor DTM resolution. Meanwhile, the Unmanned Aerial Vehicles (UAVs) survey is assured of producing highly accurate DSM/DTM, Orthomosaic, and 3D models [5]. It is an affordable and cost-effective technique for producing high-resolution DTM [[68], [69], [70]], but licensing drone flying might be tricky according to regional policies and restrictions.

**Fig. 3:**
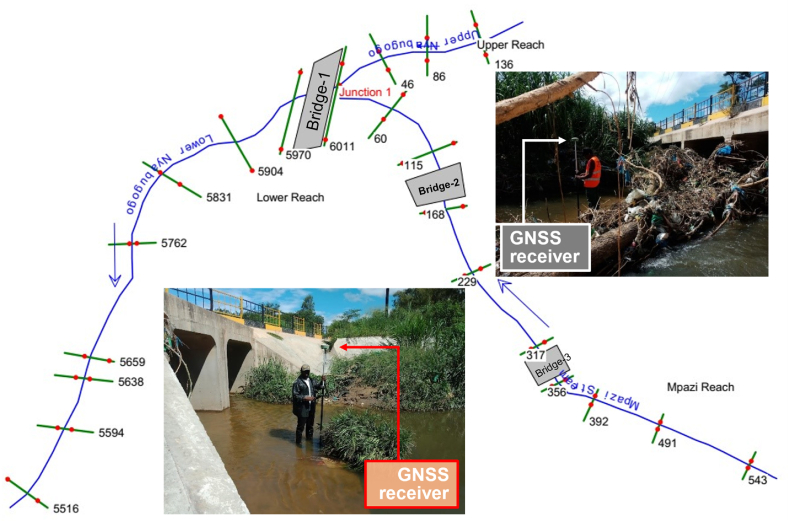
Geometric data and GNSS surveying for evaluated bridges in NRC.

The model evaluated four bridge responses to the return peak flows where Bridge-1 (Fig. 3) is one of the main bridges that connect one part of the northern and eastern regions to the main bus station in the metropolitan area. A number of coefficients were assigned in the program to evaluate respective energy losses. Manning's “n" or Equivalent Roughness “k" values for friction losses and Contraction-Expansion coefficients were all assigned according to the bridge, channel, sections, and floodplain characteristics [71]. Both the recently upgraded and not upgraded bridges were simultaneously simulated for 25-, 50-, 100-, and 150-year frequencies under ssp245 and ssp585 plans.

#### 2.5.2 Integrated hydrodynamic flow routing principles

HEC-RAS identifies the changes in energy throughout the channel using integrated Bernoulli's energy conservation equation where the outlet is the control condition (Eq. (5)) [71]. Unsteady flow routing is calculated by solving 1D/2D equations, well-known as Full Saint-Venant equations or Diffusion wave equations, derived by ensuring the principles of mass and momentum conservation balance between the two cross sections [[71], [72], [73], [74]]. A 1D Saint-Venant equations' (Eq. (6) and Eq. (7)) are a simplification of the 2D Saint-Venant equations, well known as shallow-water equations [72]. Since this study wasn't intended to simulate the flow characteristics based on direction and propagation like horizontal floodplain analysis, inundation, or surface waterlogging processes but to evaluate the bridge responses to the flow magnitudes and climate variation, the simulations were carried out with 1D hydrodynamic model under steady flow conditions. The 1D model is ideal for both steady and unsteady flow, while the 2D is ideal for unsteady flow calculations but also performs better in depicting inundations or surface waterlogging processes and floodplain characteristics.

(5) $${\text{Bernoulli}}^{\prime }s\;\text{equation},Z_{2}+Y_{2}+a_{2\;}\frac{V_{2}^{2}}{2g}=Z_{1}+Y_{1}+a_{1\;}\frac{V_{1}^{2}}{2g}+\text{He}$$

(6) $$\text{Conservation}\;\text{of}\;\text{mass},\frac{\partial Q}{\partial X}+\frac{\partial A}{\partial t}=0$$

(7) $$\text{Conservation}\;\text{of}\;\text{momentum},\frac{1}{A}\frac{\partial Q}{\partial t}+\frac{1}{A}\frac{\partial \left(\frac{Q^{2}}{A}\right)}{\partial x}+g\frac{\partial H}{\partial x}-g\left(S_{0}-S_{f}\right)=0$$

**Where** in Eq. (5), $Z_{1\&2}$ are elevation bottoms of the channel, $Y_{1\&2}$ are depths of water at the cross-section, $V_{1\&2}$ are average flow velocity, $a_{1\&2}$ are velocity weight coefficients, g is the gravitational acceleration, and He is energy head losses. In Eq. (6) and Eq. (7), X is the distance along the channel, t is the time, H is the water depth, Q is the flow rate, A is the wetted area, $S_{0}$ is the channel slope, while $S_{f}$ is the friction slope.

## 3 Results and discussion

### 3.1 GCMs downscaling and bias correction

**Fig. 4:**
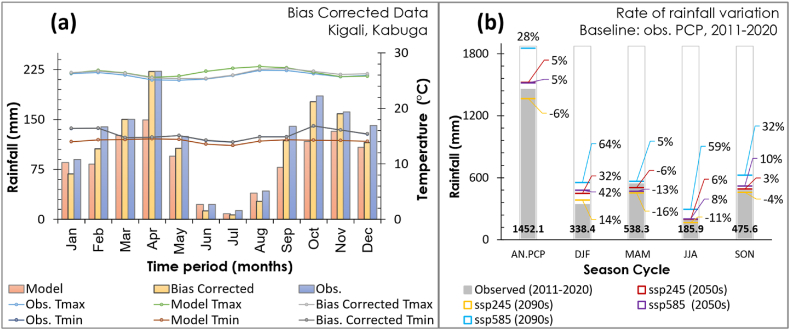
Climate projection, (a) comparison of observed and modelled data before and after bias correction, (b) rainfall variation reference to the 2010s observed rainfall.

This study used the statistical bias correction and downscaling approach [75,76]. The bias correction reanalysis was conducted using Linear Scaling, Distribution Mapping, Local Intensity Scaling methods, and Variance Scaling. However, the distribution mapping didn't perform well on precipitation and temperature data; hence, it was opted out even though it is acclaimed for a good performance in some studies [63]. Besides, Linear Scaling presents more reliability with the reference. Upon bias correction, the bias uncorrected model presented under-estimation for precipitation and minimum temperature and over-estimation for maximum temperature (Fig. 4). Afterwards, the bias-corrected data were evaluated by standard deviation and Pearson correlation coefficient. Other methods presented up to 0.96 and 0.75 correlation for precipitation and temperature in their overlapping period, respectively, while the Distribution Mapping presented less than 0.35, and the projection presented a high standard deviation (more than threefold) in comparison with all others. More insights about hydroclimatic trend analysis and projection were expressed in Ref. [24]. This study analysed the rate of rainfall variation in decadal series for the near and far future, i.e., the 2050s (2051–2060) and 2090s (2091–2100) relative to a decadal reference period, the 2010s (2011–2020) for scenarios imposed by medium and high greenhouse gas emission scenarios (ssp245 and ssp585) and the country climatic seasons described in Ref. [24]. In Fig. 4 (a), the graph presents observed and modelled data before and after bias correction in the overlapping period, while Fig. 4 (b) illustrates the rainfall magnitude (mm) with their respective rate of variation (%) at a relative climate scenario (SSP245 and SSP585) reference to the 2010s (2011–2020) observed rainfall. The bias-corrected climate presented up to 64% (+216.0 mm) and 59% (+109.1 mm) rainfall increment for December–February, and June–August dry seasons respectively, while March–May and September–November rainy seasons presented up to 5% (+27.1 mm) and 32% (+150.0 mm) rainfall increment respectively, and up to 28% (+400.5 mm) annual rainfall increment by the end-century under ssp585.

### 3.2 Model sensitivity and calibration

**Fig. 5:**
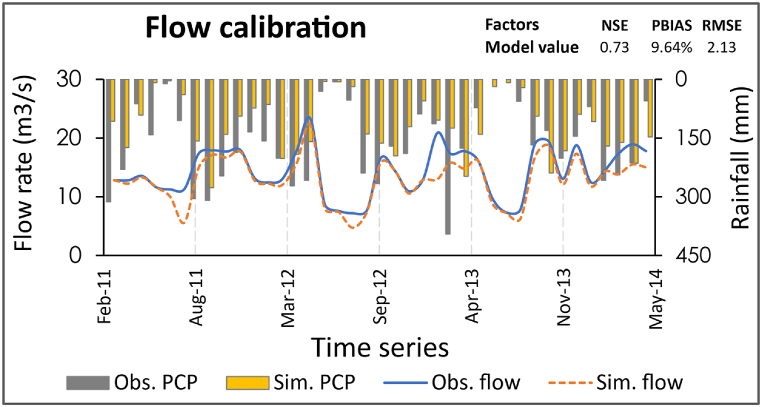
Flow calibration.

The storm designs are chosen according to the purpose of the design; for example, the irrigation schemes often choose the probable average flow rate with respect to the seasons, while the probable minimum is often an option for hydropower systems. Since this model was intended for protection structures, the daily peak flow rate was considered during calibration. As the model is built from many parameters, the study chose several hydraulic parameters to run sensitivity analysis, and the percentage change technique was used to calibrate the model (Table 3). Nine parameters found to be more sensitive were used to calibrate and validate the model through the SWAT + Toolbox. A 101-year simulation with a two-year warm-up period was run over 60 iterations. The NSE, PBias, and RMSE were used to track model performance. Mostly due to the absence or poor gouged flow data where the study had to generate the discharges with SRC (see Section 2.4.3 above), after multiple reruns through which the model is automatically calibrated and validated, it was noted that it is complex to obtain a feasible simulation as ideal as our last simulation. The model showed no further changes after 60 iterations. The study showed 0.73, 9.64%, and 2.13 for NSE, PBias, and RMSE, respectively (Fig. 5). Meanwhile, the peak flow calibration outputs slightly align with the reported calibrations in Manyifika, [60].

### 3.3 Basin peak flow analysis

**Fig. 6:**
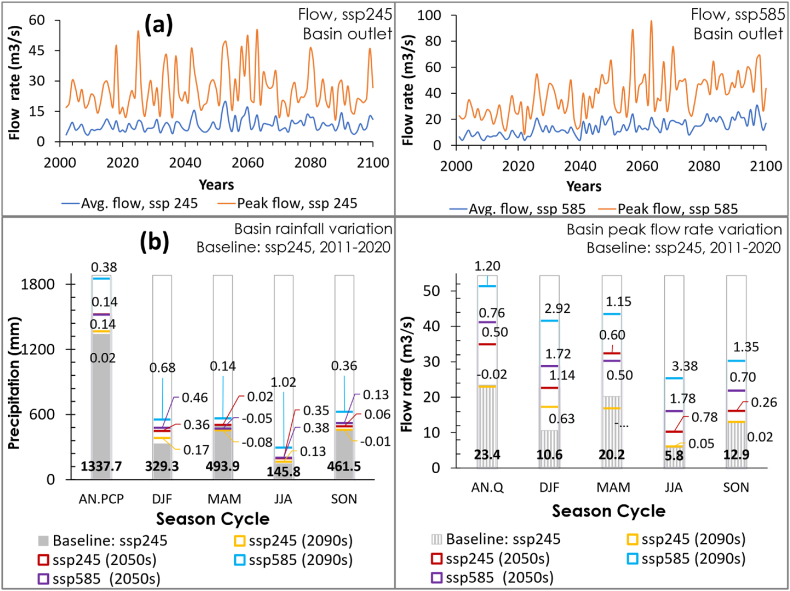
Future climate impacts on the basin flow rate variation, (a) time series, (b) rate of rainfall and streamflow variation reference to the baseline ssp245, 2011–2020.

The simulation showed up to 55.4 and 95.7 $m^{3}/s$ catchment peak flow rate under ssp246 and ssp585 respectively. The mid-century generally presents the peak flow rates under both ssp245 and ssp585 (Fig. 6,(a)). Though the precipitation increment identified in the distant future might result in the flow rate increment, the temperature increment might also result in intensified evapotranspiration, which should lead to a slight peak flow rate decline in the distant future [77]. Refer to Icyimpaye, [79] and Icyimpaye et al. [78] studies conducted on the same catchment, they obtained excess discharge because the model wasn't calibrated. More so, looking at Appendix 3 in Icyimpaye, [79], the study seemed to have an issue with poor DTM resolution, which affected the channel topography. The peak flow from the third run obtained about 180 $m^{3}/s$ in June 2018 (dry period), while the maximum stage level recorded by RWB in the whole year (two rainy seasons included) was 3.76-m during end-April, which is roughly equal to 26.6 $m^{3}/s$ by using the SRC generated in this study, and about 27.3 $m^{3}/s$ according to the SRC generated in Manyifika, [60].

The future climate impact on the hydrologic regime was also analysed in decadal series for the mid-century and end-century, i.e., the 2050s (2051–2060) and 2090s (2091–2100) relative to a decadal baseline reference, the 2010s (2011–2020) for scenarios imposed by medium and high greenhouse gas emission scenarios (ssp245 and ssp585) and the country climatic seasons described in Ref. [24]. From Fig. 6,(b), the graphs present precipitation magnitude (mm) and the streamflow rate ($m^{3}/s$) with their respective rate of variation (%) at a relative climate scenario (ssp245 and ssp585) reference to the ssp245, 2011–2020 rainfall and streamflow. The study identified up to 38% (+514.9 mm) annual precipitation increment that resulted in a 120% (+28.0 $m^{3}/s$) flow rate increment by the end of the century under ssp585. The projection revealed a vast increment in December–February, currently a cool-dry season where the precipitation and flow rate magnitude might be pretty close to the precipitation and flow rate magnitude for the March–May long rainfall season by the 2050s, mid-century (Fig. 6,(b)). Consequently, increased precipitation magnitude in this cool-dry season might result in increased Consecutive Wet Days (CWD) and rainfall frequencies, where the December–February cool-dry season and March–May rainfall season might turn out into a prolonged single rainfall season by the mid-century. But more study is recommended. As a result, the study showed that up to 68% (+225.1 mm) precipitation increment resulted in 292% (+31.0 $m^{3}/s$) flow rate rise in the December–February cool-dry season by mid-century (the 2050s) under ssp585, while a doubled precipitation (+149.2 mm) resulted in a 338% (+19.6 $m^{3}/s$) flow rate increment in June–August by the end of the century (the 2090s) under ssp585, which is currently a warm-dry season. Thus, as a country severely affected by deadly landslides and rainfed agriculture [24,26,27], climate mitigation and pre-emptive adaptation are highly recommended in agriculture, transportation, and water-related infrastructure development.

Besides, referring to Umugwaneza et al. [80], a study conducted on average annual discharge in the same basin, the study reported a 3.26% increase in 2020–2050 and a 4.53% decline in 2050–2100 under ssp585. However, the study used neither statistical nor dynamic bias correction technique but used IDW in downscaling or interpolating precipitation to a desired station, which should still give out the mother model resolution output as fine as 25–50 km for RCMs and more than 111 km for GCMs [81]. In case higher resolution climate information is needed, the RCMs may not be suitable [81]. Moreover, evaluating the changes over about 6-decades as a single timespan may not provide a clear image of the time series fluctuation impacts. Meanwhile, the effects of extreme future flow rates induced by excess precipitation have been identified globally. According to Ye et al. [82], the study identified up to 400% and 18% flow rate increments resulting from rainfall variation and land use/cover change, respectively, by the end of the century. The study conducted by Li et al. [77] identified up to 162.47% variation by end-century under ssp245, while the study conducted by Haider et al. [83] identified up to 345.3% change factor under RCP8.5.

### 3.4 Bridge storm design and frequency analysis

**Fig. 7:**
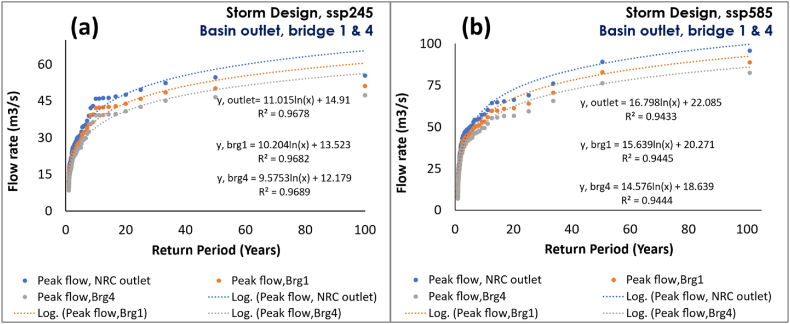
Storm designs and peak flow frequencies for the catchment and study bridges under, (a) medium GHG emission scenario, (b) high GHG emission scenario.

This study identified up to 38% (+514.9 mm) annual precipitation increment, which resulted in up to a double flow rate increment (+28.0 $m^{3}/s$) by the end of the century under ssp585 (Fig. 6. (b)). For a 100-year bridge storm design and frequency analysis, the study shows that the peak flow rate at the basin outlet might increase up to 55.4 and 95.7 $m^{3}/s$ under ssp246 and ssp585, respectively, and increases up to 47.4 and 82.4 $m^{3}/s$ under ssp246 and ssp585, respectively, at Bridge-4 (Fig. 7). Looking at studies conducted in the region [24,26,27], the studies reported up to 29% precipitation increment reference to the current period. This rainstorm increment would obviously translate to a rise in the streamflow rate in the hydrologic regime. The study by Rukundo & Doğan [28], conducted using the HadCM3 model of IPCC-AR4/CMIP3 [7,8,84], reported 73.4 up to 86.6 $m^{3}/s$ peak discharges for a 100-year storm, which slightly aligns with the ssp585 results in this study (Fig. 7). However, despite an excess precipitation increment reported in the climate projection study done using different CMIP3 GCMs [27], the study by Rukundo & Doğan [28], reported a flow rate decline up to 39.9 and 41.1 $m^{3}/s$ for 100-year return under HadCM3A2 and HadCM3B2 climate projection scenarios respectively. A flow rate discrepancy that might have resulted from HadCM3, CMIP3 model deficiencies [[85], [86], [87]], and the use of a single model while a multi-model ensemble (MME) is acclaimed to be more reliable than the best single model [40,41].

### 3.5 Bridge response to the future climate

Infrastructures are designed for a specific lifespan and are expected to be useful throughout their lifespan, which counts to 100 years for bridges and drainage systems. Apart from different types of loadings, the bridge design undertakes a number of external factors, such as geophysical dynamics and hydrodynamics, i.e., the weather-related impact, to ensure the structure is satisfactory and safe throughout its lifespan [3,88,89]. A clear knowledge of future hydroclimatic state is obvious for a reliable design. So, the main objective for future climate modelling presented above in this study was to assist in evaluating how the current hydraulic structures might respond to future climate variations. The study evaluated the bridges' responses to future climate peak storms. This work considered four bridges (Fig. 1) that are more vulnerable to climate change impacts. The storm frequencies and non-exceedance probability were analysed from the Normal cumulative distribution function (CDF) developed based on a 100-year flow rate simulation. Both the bridges were analysed for 25-, 50-, 100-, and 150-year return peak storms under ssp245 and ssp585 scenarios. Upon the simulation, the bridges responded differently to the future climate. The two bridges along the Mpazi stream overstood future climate peak storms under both medium and high GHG emission scenarios, while two other bridges along the Nyabugogo River failed to accommodate 50-year peak storms under high GHG emission scenarios.

**Fig. 8:**
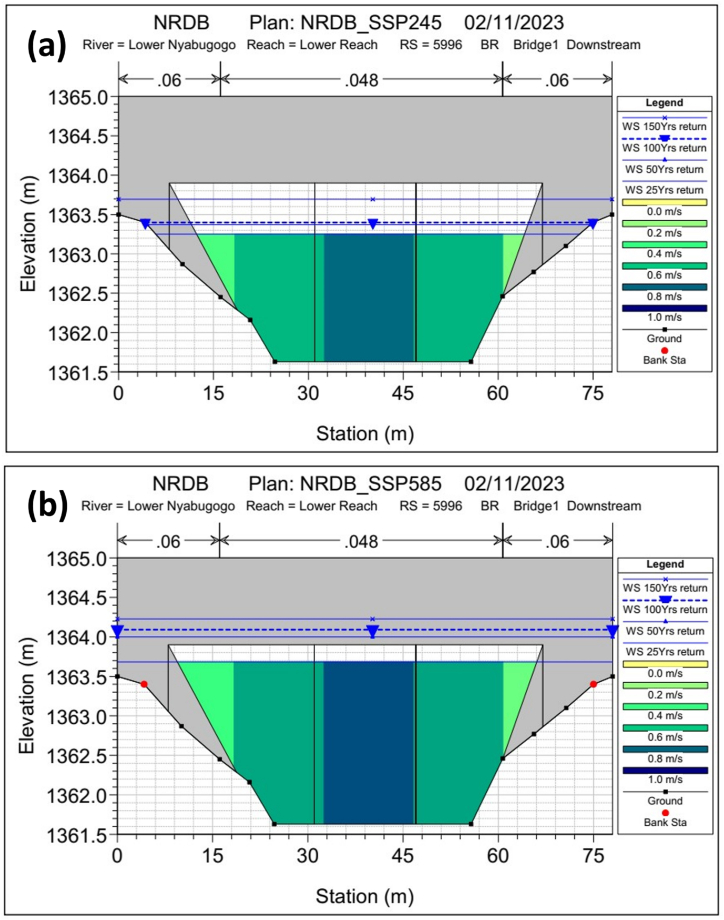
Bridge-1 response to the climate change under, (a) medium GHG emission scenario, (b) high GHG emission scenario.

Upon the simulation, the Bridge-1 located at Nyabugogo lower channel (Fig. 3) presented about 0.5-m freeboard depth for 100-year return peak flow, and 0.21-m for 150-year return under ssp245, and presented 0.22-m freeboard depth on 25-years return peak under ssp585 but overtopped at the 50-years return peak storm (Fig. 8). In essence, the Bridge-1 showed a satisfactory performance up to the end-century (the 2090s) under ssp245 and unsatisfactory performance up to the mind-century (the 2050s) under ssp585; which implies that this main bridge in the catchment might be safe throughout its lifespan under medium Greenhouse Gas (GHG) emission scenario, but it can't accommodate flood-induced climate change impacts under high GHG emission scenarios throughout its lifespan. Henceforth, the high GHG emission scenarios are recommended to be considered for critical infrastructure development. Besides, low-impact development, best management practices (LID-BMP), or green-grey infrastructures have been widely studied and have proven excellent performance in urban drainage and stormwater management and quality control [5,[90], [91], [92], [93]]. Therefore, enforcing these approaches would enhance the drainage and stormwater management in the region. However, further studies are recommended to ensure proper performance at each of the respective approaches. Besides, the fossil fuels burning reduction and enforcing clean energy sources remain ideal mitigation measures for climate change effects.

### 3.6 Study limitation

The streamflow analysis with hydrological models incorporates several factors that come with a range of uncertainties. Hydroclimatic impacts, which were addressed in this study, are the main source of uncertainty. This study used the most recent Esri 10-m resolution land use/cover classes but remained static in the runoff quantity calculations due to the resources and time limitations. Although the LULC generally has a significant impact on the rainfall-runoff simulations [63] and obviously won't be the same in the future, the variations in hydroclimatic patterns are the main factors in runoff quantity calculations. Meanwhile, the future land use projections are simulated based on algorithmic modules like artificial neural networks (ANN) or cellular automata (CA) theory that endeavours to recognise a complex relationship underlying between land use/cover patterns and human activities or environmental factors which are often extracted from remotely sensed imageries [63,[94], [95], [96]].

## 4 Conclusion and recommendations

### 4.1 Conclusion

This study evaluated the future climate impacts on hydraulic infrastructure development in a tropical (peri-) urban catchment from Kigali, Rwanda. The study findings present excessive precipitation increments, which might result in increased extreme flood and landslide events. The study identified up to 38% (+514.9 mm) annual precipitation increment under a high greenhouse gas emission scenario that resulted in a doubled flow rate increment (+28.0 $m^{3}/s$) by the end of the century. The projection revealed a huge increment in December–February, currently a cool-dry season where the precipitation and flow rate magnitude might be quite close to the precipitation and flow rate magnitude for the March–May long rainfall season by the 2050s, mid-century (Fig. 6,(b)). As a result, hydrodynamic simulation revealed that some bridges in the catchment might fail to accommodate the 50- and 100-year peak storm where, for instance, Bridge-1 presented about 0.5-m freeboard depth for 100-year return peak flow under ssp245 and 0.22-m freeboard depth on 25-years return peak under ssp585 but overtopped at the 50-years return peak storms; which implies that this bridge might be safe throughout its lifespan under medium greenhouse gas emission scenario, but it can't accommodate flood-induced climate change impacts under high greenhouse gas emission scenarios throughout its lifespan. Besides, the two bridges along the Mpazi stream overstood the peak flows under both high and medium greenhouse gas emission scenarios.

Furthermore, based on the study findings, the literature [23,26,31,53,[97], [98], [99], [100]], and the topographic characteristics, the flooding in Kigali seems to be the result of intense rainfall, drainage sizes, urbanisation, plus the topography where a huge part of Kigali metropolitan area resides in a lower altitude area of the basin (Fig. 2), making it more subjected for the floods induced by heavy rainfall from this basin. Henceforth, there is a need to adopt high GHG emission scenarios in critical infrastructure development, the necessity to adopt climate adaptive construction practices (green-grey infrastructure), and integrate the use of hydrologic-hydrodynamic, risk, and life cycle cost-benefit analysis for resilient infrastructure provision and financing. Thus, the results of this study might prove useful in climate-resilient infrastructure development and other pre-emptive adaptation practices, most importantly building anticipated resilience against climate-related hazards.

### 4.2 Recommendations

There have been various reports claiming bridge structures swept away by heavy rainfall coupled with massive landslides mostly stirred up by a sloppy topography of the country, a mountainous region fragile to erosion and landslides. The impacts of stormwater lateral force and turbulence accelerated by topography result in the bridge's total failure. Moreover, the flow velocity and turbulence with unsettled eroded materials are the main driving factors for abutment scours, which result in the bridge failure processes [101,102]. Henceforth, studies analysing the impacts of future climate peak stormwater lateral force on the bridge dynamics in this region are recommended. More so, enforcing green-grey infrastructures in flood risk-low resilient areas is equally recommended to improve resilience against climate risks. Further, Some more sources of uncertainties were recommended for future research and practical implications.

• Advanced techniques in acquiring gouged flow data in this basin are highly recommended.
• Further study applying a larger ensemble, high-resolution soil data, future land use/cover, different bias correction techniques, and different calibration techniques is recommended.
• The use of LiDAR- or UAV-based topographic data and drainage hydraulic performance in hydrodynamic simulations is recommended.

## 5 Data availability statement

The data will be made available on request through a corresponding author.

## CRediT authorship contribution statement

**Parfait Iradukunda:** Writing – review & editing, Writing – original draft, Visualization, Validation, Resources, Methodology, Investigation, Formal analysis, Data curation, Conceptualization. **Erastus M. Mwanaumo:** Supervision, Resources, Project administration, Funding acquisition, Conceptualization. **Joel Kabika:** Supervision.

## Declaration of competing interest

The authors declare that they have no known competing financial interests or personal relationships that could have appeared to influence the work reported in this paper.
